# Role of Tumor-Associated Macrophages in Cervical Cancer: Integrating Classical Perspectives with Recent Technological Advances

**DOI:** 10.3390/life14040443

**Published:** 2024-03-27

**Authors:** Yeseul Choi, Donghyeon Lee, Na Young Kim, Incheol Seo, Nora Jee-Young Park, Gun Oh Chong

**Affiliations:** 1 Graduate Program, Department of Biomedical Science, School of Medicine, Kyungpook National University, Daegu 41944, Republic of Korea; yeseul.choi830@knu.ac.kr (Y.C.); lowellkids24@knu.ac.kr (D.L.); skduddl98@knu.ac.kr (N.Y.K.); 2 Department of Immunology, School of Medicine, Kyungpook National University, Daegu 41944, Republic of Korea; iseo@knu.ac.kr; 3 Clinical Omics Institute, Kyungpook National University, Daegu 41405, Republic of Korea; pathpjy@knu.ac.kr; 4 Department of Pathology, Kyungpook National University Chilgok Hospital, Daegu 41404, Republic of Korea; 5 Department of Obstetrics and Gynecology, Kyungpook National University Chilgok Hospital, Daegu 41404, Republic of Korea

**Keywords:** cervical cancer, tumor-associated macrophage, M1-like phenotype, M2-like phenotype, angiogenesis, metastasis, multi-omics, single-cell transcriptomics, spatial transcriptomics

## Abstract

Tumor-associated macrophages (TAMs) play a pivotal role in the tumor microenvironment, influencing cancer progression and contributing to poor prognosis. However, in cervical cancer (CC), their significance and involvement are relatively less studied than in other gynecological cancers such as ovarian and endometrial cancer. This review aims to provide an overview of TAMs, covering their origins and phenotypes and their impact on CC progression, along with major TAM-targeted therapeutic approaches. Furthermore, we advocate for the integration of cutting-edge research methodologies, such as single-cell RNA sequencing and spatial RNA sequencing, to enable in-depth and comprehensive investigations into TAMs in CC, which would be beneficial in leading to more personalized and effective immunotherapy strategies for patients with CC.

## 1. Introduction

It has been widely acknowledged that tumor microenvironment (TME), which comprises diverse cell types such as tumor, immune, and stromal cells, plays a critical role in cancer progression. Many studies have investigated complex reciprocal communications between TME cell components, especially the involvement of immune cells within the TME. Macrophages, as a key myeloid immune cell type, are responsible for front-line defense as well as providing the innate immune response [1]. Notably, macrophages are the major infiltrated immune cell types in the TME, representing 50–70% of its components, and are known as tumor-associated macrophages (TAMs) [2].

Cervical cancer (CC), along with ovarian and endometrial cancer, is a major gynecological cancer. Given their substantial presence and impact within the TME, the investigation and further knowledge of TAMs in CC is invaluable. However, research on TAMs in CC remains relatively limited compared with that in ovarian or endometrial cancer. 

Thus, this review aims to emphasize the importance of TAMs in CC, providing an overview of their origin, polarization, and role in CC progression. Additionally, we assess the necessity of leveraging recent advanced research technologies such as single-cell RNA sequencing (SC RNA-seq) and spatial RNA sequencing (SP RNA-seq). These cutting-edge techniques would allow for in-depth and novel findings regarding TAMs in CC, the discovery and redefinition of current understandings of TAMs in CC, and the further development of TAM-targeted therapeutics for patients with CC. 

For this narrative review, an extensive literature search was conducted utilizing databases including PubMed, Science Direct, Elsevier, and Google Scholar. Electronic retrieval focused on studies pertaining to TAMs, encompassing terms such as ‘Tumor-associated macrophage’, ‘TAMs’, ‘M1’, ‘M2’, ‘TAM polarization’, and ‘Cervical cancer’. Additionally, literature related to keywords such as ‘Hypoxia’, ‘TAM targeted therapy’, ‘Single-cell RNA seq’, and ‘Spatially RNA-seq’ was gathered. The search predominantly targeted publications released between January 2017 and December 2023. A few highly influential articles predating 2017 were also included due to their significance to the subject matter. 

## 2. Origin and Polarization of TAMs

### 2.1. Origin of TAMs

Two origins explain the presence of macrophages in tumors: tissue-resident macrophages (TRMs) and tissue-infiltrating macrophages (TLMs). TRMs derive from the yolk-sac during embryonic organogenesis and differentiate into tissue-specific phenotypes and are activated by following their organ-specific purpose [3]. Conversely, TLMs originate from bone marrow. Inflammatory signals from tumors and the TME recruit circulating monocytes that differentiate into TLMs [4]. 

TAMs are a subset of TLMs that specifically accumulate in the TME [5]. TAMs have high plasticity and differentiate into different phenotypes depending on the contexts, signals, and environmental cues within the TME. Broadly, TAMs are characterized into the classically activated M1-like type, which is involved in pro-inflammatory functions, or alternatively activated M2-like types, which are involved in pro-tumoral functions [6,7].

### 2.2. Classical Concept of TAM Polarization

M1-like TAMs are induced by T-helper 1 cytokine interferon-γ (IFN-γ), lipopolysaccharide (LPS), or Toll-like receptor (TLR) agonists [8]. These primarily engage in proinflammatory functions by secreting pro-inflammatory cytokines such as interleukin 1 (IL-1), IL-6, tumor necrosis factor-α (TNF-α), reactive oxygen species (ROS), or nitric oxide (NO) [9]. M1-type TAMs rely on glycolysis for their metabolism and are characterized by a high glutathione level and iron retention [10]. Altogether, M1-like type TAMs contribute to pro-inflammatory responses, pathogen clearance, microbicidal activity, and anti-tumoral functions.

**Figure 1:**
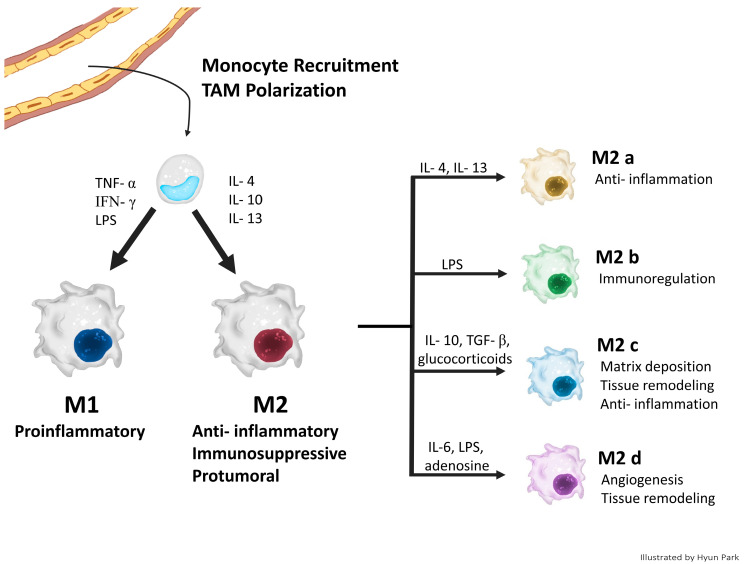
The classical concept of M1 and M2 TAM polarization.

The alternatively activated M2-like-type TAMs are mainly associated with pro-tumoral activities such as anti-inflammatory and immune-suppressive functions [11,12]. Depending on the signals they receive, the M2-like TAMs further differentiate into four subtypes with distinct phenotypes and functions: M2a, M2b, M2c, and M2d [8]. M2a TAMs are activated by T-helper 2 (Th2) cytokines such as IL-4 or IL-13 and secrete anti-inflammatory cytokines such as IL-10 and transforming growth factor (TGF-β) that elevate the expression of vascular endothelial growth factor (VEGF) or matrix metalloproteinases (MMPs) [8]. M2b is induced by LPS or through TLR ligands, releasing cytokines such as TNF-α, IL-1β, IL-6, IL-10, or CCL1, which contribute to Th2 activation and immunoregulation [8]. M2c becomes activated by receiving IL-10, TGF-β, and glucocorticoids, and primarily participates in matrix deposition, tissue remodeling, and anti-inflammation by secreting IL-10, TGF-β, C-C motif ligand 16 (CCL16), CCL18, and CXC chemokine ligand (CXCL13) [13]. Lastly, M2d is induced by adenosine, LPS, IL-6, and leukemia inhibitor factor (LIF) elevating the levels of VEGF, TGF-β, and IL-10,while decreasing TNF-α or IL-12 levels, thereby promoting tissue remodeling and angiogenesis [13]. The metabolism of M2 types involves oxidative phosphorylation and fatty acid oxidation with low glutathione and iron release [10]. The classical concept of M1 and M2 TAM polarization and four subtypes of M2 TAMs are summarized in Figure 1.

TAMs have been proposed to be more skewed toward M2 features, and monocytes polarize into M2-like TAMs through various signals within the TME, a process termed TAM polarization. For example, the CCL2/MCP-1 axis activates nuclear factor kappa β (NF-kβ) signaling, inducing inflammation and cancer initiation while modulating macrophages into M2-like phenotype TAMs [14] Furthermore, IL-6 inhibits the original phagocytic function of macrophages via the JAK/STAT3 pathway [15], while IL-10 elevates arginase expression and induces M2-like immunosuppressive activity in macrophages [3]. 

Several studies have also found that TAM polarization toward M2-like features is more closely associated with CC. Tan et al. discovered that the c-myc/PKM2 pathway upregulates IL-10 secretion, thereby inducing TAM polarization toward M2 types, which was positively correlated with adverse outcomes such as tumor growth and the metastasis of CC [16]. Furthermore, coculturing macrophages with supernatants of CC induced polarization toward the M2 type [17]. Li et al. found higher intratumoral and peritumoral M2 TAM density in CC tissue samples compared with nontumorous cervical tissue [18]. 

## 3. The Role of TAMs in CC Progression

TAMs play crucial roles in nearly every aspect of tumorigenesis and cancer progression [19,20,21,22]. Their involvement in cancer progression can be elucidated by the concept of hallmarks of cancer [23]. Herein, we review how TAMs participate in CC progression via major hallmarks of cancer.

### 3.1. Inflammation/Initiation

Broadly, inflammation related to cancer arises from two pathways. The first is intrinsic, driven by genetic mutations or alternations, whereas the other is extrinsic, triggered by inflammatory conditions at specific anatomical sites [24]. The convergence of these two pathways activates signals such as NF-kβ and hypoxia-inducible factor (HIF-1α) with STAT3 in tumor cells, which causes monocyte recruitment and the production of pro-inflammatory signals such as IL-10 or TGF-β in the TME via M2-like TAMs [15,24]. 

### 3.2. Angiogenesis

TAMs are also involved in the angiogenesis of CC [25]. TAMs tend to be accumulated and be enriched in hypoxic TMEs with poor blood supply [26]. Hypoxia, a hallmark of cancer, intricately interacts with TAMs, influencing macrophage recruitment, macrophage entrapment, and polarization toward M2-like phenotypes [27]. First, hypoxia attracts macrophages to the TME through various chemoattractants such as CCL2, CCL5, or CSF-1 [27]. Subsequently, it impedes macrophage mobility, culminating in TAM accumulation within the hypoxic niche. Then, various factors including CSF-1, TGF-β, and ILs gradually polarize the entrapped macrophages toward M2-like TAMs [14]. 

The heightened presence of TAMs in hypoxic TME further induce and augment angiogenesis by upregulating proangiogenic genes like HIF-1 and secreting proangiogenic factors such as VEGF, fibroblast growth factor (FGF), platelet-derived growth factor (PDGF), MMPs, IL-1, IL-8, TNF-α, and NO [28,29]. TIE 2, an angiopoietin (ANG) receptor expressed by TAMs is also upregulated under hypoxic conditions and further amplifies the expression of proangiogenic genes such as MMP9, VEGF, COX2, and WNT5A when combined with ANG-2 [30]. These molecules collectively promote endothelial cell proliferation, matrix remodeling, and aberrant vascularization in TME [29,31]. These changes increase vascular density and can even promote the hypoxic TME through positive feedback, thus accelerating angiogenesis. 

In CC, the hypoxic TME recruits macrophages and causes TAM polarization toward the M2-like phenotype, as well as TAM infiltration via the Nrp-1 axis [32]. Also, an increased number of TAMs has been reported to positively correlate with higher microvessel density, indicating a higher potential of tumoral inflammation and lymphangiogenesis in CC [33,34]. Ding et al. found that macrophages cocultured with CC cells exhibited upregulated mRNA expression of VEGF-A and VEGF-C compared with that in a single culture of macrophages without CC cells, suggesting the active involvement of TAMs in lymphangiogenesis in CC via the interaction with CC tumor cells [35]. Chen et al. also discovered that a hypoxic niche promotes the formation of lymphatic vessels encapsulated by TAMs, causing lymphoangiogenesis [36]. Furthermore, they found that the number of hypoxic TAMs and lymphatic vessel density were significantly higher in lymph node metastasis (LNM) tissue of cervical squamous cell carcinoma (CSCC) than that in non-LNM tissue of CSCC, suggesting the significant involvement of TAMs in angiogenic features of CC that may cause metastasis and poor prognosis [36]. 

### 3.3. Invasion/Migration/Metastasis

TAMs also play a critical role in the invasion of CC tumor cells, as well as CC metastasis, from preparing the premetastatic niche to recruiting circulating tumor cells to the metastatic site and further fostering tumor growth [37]. Once TAMs are recruited and located at the perivascular or at the invasive edge of the tumor, they secrete promigratory factors such as epidermal growth factor (EGF), which facilitates the proteolytic remodeling of the extracellular matrix (ECM) and accelerates tumor motility [37]. TAMs also secrete MMPs such as MMP2 and MMP9, which degrade the ECM, leading to metastasis to circulatory or lymphatic systems, and promote the epithelial–mesenchymal transition (EMT) process [38,39]. Zhu et al. also discovered that high-risk human papillomavirus (HPV) oncoproteins induce metastasis via MMPs [40]. Moreover, TGF-β expressed by TAMs induces the EMT of tumor cells, leading to invasion and metastasis [38]. TAM-produced chemokines such as CCL2 are also known to positively correlate with TME invasion and regional metastasis [41]. 

TAM density itself is significantly higher in patients with CC with distant metastasis [42]. Jiang et al. discovered that TAM density and the number of tumor neovessels gradually increased in proximity along with CC invasion progression and argued that TAMs and tumor neovessels together promote CC invasion [43]. Chen et al. used *The Cancer Genome Atlas* to show that hypoxia-induced ZEB1 fosters a prometastatic TME by secreting CCL8, which recruits TAMs via NF-kβ signaling [44]; they further argued that the increased expression of ZEB1 in CC was significantly correlated with LNM and an advanced Federation of Gynecology and Obstetrics (FIGO) stage, indicating the involvement of TAMs and a TAM-dependent manner of CC metastasis and progression.

### 3.4. Immunosuppression

TAMs in the TME also secrete various factors that cause an immunosuppressive TME. Cytokines such as CCL17 and CCL15 expressed from TAMs attract regulatory T cells (T regs) into the tumor stroma, whereas IL-10 and TGF-β suppress T-cell-mediated anti-tumor immune responses [45,46]. TAMs also inhibit the apoptotic function of natural killer cells and further induce the conversion of CD 4+ T cells into T-regs, enhancing their immunosuppressive functions [38]. Additionally, TAMs inhibit the recruitment of CD 8+ T cells into the TME via CXCL9 and CXCL10 [47]. Meanwhile, IFN-γ or TNF-α from TAMs upregulate the PD-L1 expression of tumor cells, and IL-10 and TGF-β from TAMs influence the B7-H1 expression of tumor cells. The increased expression of PD-L1 and B7-H1 enables binding to the PD-1 receptor of T cells and dampens the cytotoxic activity of T cells, thereby allowing for escape from immune surveillance [48]. 

Ring et al. found the presence of both TAMs and T regs in CC tumor cells [49]. Moreover, they further suggested TAMs as the main contributor of an immunosuppressive TME, with evidence of a higher density of intratumoral TAMs over T regs [49]. Another study found that the mesenchymal and stromal cells of CC have a greater potential to promote the M2 polarization of TAMs, as well as inhibiting T cell proliferation and promoting FoxP3+ T reg subsets, leading to immunosuppression [50]. Heeren et al. emphasized the critical role of PD-L1 in patients with cervical adenocarcinoma with poor disease-free survival (DFS), indicating its role in immune escape and its adverse effect in CC [51]. 

### 3.5. Cancer Stem Cells

Cancer stem cells (CSCs) are defined as a subset of tumor cells with highly tumorigenic properties such as self-renewal and differentiation potential, which ultimately contribute to chemoradiotherapy resistance and tumor initiation [52]. Their pivotal role in various facets of tumor progression is underscored by their close interplay with TAMs, of which CSCs actively foster the induction, maintenance, and expansion of CSC populations within the TME [21,53]. 

This intricate interaction forms a positive feedback loop that reinforces the protective niche to CSC survival and stemness. To illustrate this, CSCs orchestrate the recruitment of macrophages into the TME and differentiate them into TAMs. Subsequently, TAMs construct niches that are favorable for CSC survival and the maintenance of CSC stemness TME through the secretion of chemokines such as CCL2, CCL3, or CXCL12, alongside ILs and CSF-1 [52]. Furthermore, CSCs facilitate the polarization of macrophages in the TME by releasing secreting chemokines, ILs, or through exosomal communication [52]. Lastly, TAMs sustain and enhance CSC stemness, primarily via EMT by secreting CC and CXC cytokines, IL-6, IL-8, TNF-α, and TGF-β [54]. 

Many studies have investigated TAM–CSC interactions across various cancer types. Notably, in hepatocellular carcinoma and breast cancer, TAMs secrete IL-6 and activate the pro-oncogenic JAK/STAT3 pathway and WNT pathway, thereby promoting chemoresistance and invasiveness [55,56]. In oral squamous cell carcinoma, TAMs contribute to CSC-like characteristics and the maintenance of stemness through the production of chemokines like CXCL8 and CXCL12 [21]. Despite these insights, the specific dynamics of CSC–TAM interactions remain relatively underexplored in CC. 

### 3.6. Prognosis

Similar to other cancer types, TAMs are mainly associated with poor prognosis in patients with CC [46,57,58,59,60]. For example, high numbers of CD68+ macrophage infiltration were found in CC tissues, and their counts linearly increased with CC progression [61]. Wang et al. found that all macrophage phenotypes, M0, M1, and M2, were enriched in the stromal components of CC [62]. Den Boon et al. found a gradual increase in intraepithelial CD68+ and CD163+ macrophage infiltration from HPV infection and CIN to invasive CC progression [63].

However, most research indicates that M2-like TAMs are more related to negative CC prognosis. For example, a high density of M2-like TAMs was significantly correlated with a poor response to chemo- and radiation therapies, shorter DFS, and shorter overall survival [64,65]. Additionally, a high density of CD163+ macrophage was correlated with higher PD-L1 expression in tumor cells and poor clinical outcomes such as advanced FIGO stages and shorter recurrence-free survival; but the same effect was not observed in CD68+ macrophages [48,66]. Chen et al. also argued that although both CD68+ and CD163+ TAMs were positively correlated with patients with CC and high-risk HPV infection, CD163+ TAMs were significantly associated with higher FIGO stages and poor prognosis compared with CD68+ macrophages [67]. Guo et al. supported this trend by stressing that although both CD68+ and CD163+ TAM infiltration in CC were associated with tumor progression, CD163+ M2-like TAM infiltration was associated with more advanced FIGO stages and LNM in CC [68]. Dimitrova et al. recently asserted that intratumoral and stromal CD68+ TAM infiltration have no association with different FIGO stages or lymph node involvement and are not suitable for prognosis prediction [69]. 

## 4. TAM-Targeting Therapy

Current TAM-targeting therapies have been actively developed for many solid tumors [4,70,71,72]. Many studies have used therapeutic methods in clinical trials for ovarian cancer [73,74,75]. However, TAM-targeting therapeutic methods that directly deal with patients with CC remain limited. Acknowledging their importance and significance, here, we briefly introduce major TAM-targeting therapeutic methods used in solid cancers that may also be applicable to CC. 

### 4.1. Depletion of TAMs

One of the primary TAM-targeting therapies is direct depletion of TAMs. For example, Trabectedin targets TAMs by inducing their apoptosis through activating extrinsic apoptotic-signaling pathways such as Fas and TNF-related apoptosis-inducing ligand receptors with caspase 8 [24]. Trabectedin is known to selectively reduce the number of TAMs without affecting other lymphocytes within the TME and has received FDA approval for the treatment of metastatic liposarcoma and leiomyosarcoma [76]. Engineered liposomes containing M2-like TAM-targeting components can also effectively target and delete TAMs [77]. Lastly, the use of bisphosphonates such as the biophosphonate–glucomannan conjugate is another example of direct targeting and the elimination of TAMs [78]. In a mouse sarcoma model, researchers observed decreased angiogenesis following TAM depletion, thereby validating the efficacy of TAM-targeted immunotherapy [78]. 

### 4.2. Inhibiting Monocyte/Macrophage Recruitment

Another prominent therapeutic strategy is inhibiting TAM recruitment into the TME. For example, as CCL2 attracts monocytes into the TME and converts them into TAMs, blocking the CCL2/CCR2 axis suppresses TAM migration and restrains TAM differentiation [79]. Notably, Engeletin treatment was shown to limit monocyte recruitment to the TME and suppress angiogenesis in CC cells by inhibiting the secretion of CCL2 via the blockade of the NF-kβ signaling pathway [80]. 

Inhibiting the CSF/CSF-1R pathway also decreases the recruitment of TAMs and as their polarization [79]. For example, BLZ945, a CSF-1 inhibitor is being applied to patients with glioblastoma, pancreatic cancer, or breast cancer [15,81]. Hindering these axes not only prevents TAM recruitment but also enhances the cytotoxic immune response functions of T cells and improves therapeutic efficacy [82,83]. Several clinical trials targeting TAMs via the CSF/CSF-1R pathway in CC have been registered, including NCT04542356, NCT04514692, and NCT03206684 [15]. NCT04542356, a clinical trial that evaluated the efficacy and safety of PEG-rhG-CSF during chemoradiotherapy in patients with CC, concluded its effectiveness upon its completion in 2021 [84]. 

### 4.3. Reprogramming/Re-Education of TAMs into M1-like Phenotype

The main concept of the re-education of TAMs is to attenuate M2-like TAM abilities and facilitate a pro-inflammatory and anti-tumoral M1-like phenotype. Some methods aim for a direct reprogramming of TAMs M1-like features through activating TLR signaling or inducing the cytotoxic effect of cancer cells via IFN-γ treatment [85,86]. Another method is to exploit immune checkpoint blockades. PD-L1 is strongly expressed on TAMs to induce T-cell exhaustion and immune escape as a consequence [9]. Therefore, blocking the PD-1/PD-L1 axis would remodel TAMs by reversing immunosuppressive features and mainly sustaining the pro-inflammatory functions of the M1-like phenotype [87,88]. 

CD47–SRIPa inhibition is another example of TAM reprogramming. Tumor cells highly express CD47, a transmembrane glycoprotein, on their surface. Together with a signal regulatory protein alpha (SRIPa), CD47–SRIPa exerts a “don’t eat me” signal that restricts the phagocytosis of macrophages [89,90]. Therefore, disrupting the CD47/SRIPa axis via monoclonal antibodies switches and re-educates TAMs from the tumor-promoting phenotype into the pro-inflammatory M1-like phenotype with enhanced phagocytotic behavior [91,92]. One of the major oncogenes of cancer, C-myc, amplifies the expression of PD-L1 and CD47 [93], indicating the significance of prioritizing these axes for TAM-targeting therapy. 

### 4.4. Other Methods 

Besides the main TAM-targeting therapy approaches, various innovative methods have also been used to target TAMs. For example, chimeric antigen receptor (CAR) engineered T cells have been designed to specifically target and remove TAMs [94,95]. Building upon this technique, Klichinsky et al. discovered that CAR macrophages (CAR-Ms) therapy decreased the tumor burden and prolonged survival time by inducing pro-inflammatory cytokines and chemokines into the TME and converting M2-like macrophages to M1-like macrophages [96]. Nanoparticles loaded with specific antibodies or therapeutic agents are being utilized for the improved specificity of targeting and inducing therapeutic effects directly into TAMs [97,98,99]. M1-derived exosomes, microRNA modulations, and cancer vaccines are also being developed [100,101]. 

Another strategy is to target hypoxic TME, alleviating hypoxia within the TME. Hypoxia-targeting therapy methods involve direct delivery of oxygen to hypoxic area via nanoparticles, the normalization of tumor vasculature, or the modulation of the HIF signaling pathway [30]. In CC, Tirapazamine was investigated as a potential drug targeting hypoxia; however, clinical and preclinical studies did not demonstrate significant efficacy [102]. 

## 5. Novel Findings of TAMs in the TME with Recent Technologies: Multi-Omics, SC RNA-Seq, and SP RNA-Seq

In the pursuit of a comprehensive and in-depth understanding of this topic, research increasingly employs multi-omics analysis. This approach encompasses the investigation of the target from various levels, including DNA, RNA, proteins, or microbiomes, with the aim of integrating findings from each level to construct a holistic interpretation. Recent additions to this approach are SC RNA-seq, which investigates gene expression at the single-cell level, and SP RNA-seq, which incorporates spatial and location-specific information along with the transcriptomics profile [103,104,105]. These advanced technologies are actively being applied to study TAMs in solid tumors, including CC [106,107,108,109,110,111,112,113,114]. 

Notably, these technological advancements are unveiling unexpected and novel insights into TAMs in cancer, challenging the traditional dichotomous classification of TAMs into M1- and M2-like phenotypes. Conversely, they reveal the coexistence of M1- and M2-like phenotypes within the TME and further assert that TAMs exist as an extended spectrum between M1 and M2, encompassing several distinct types. 

For instance, recent studies employing SC RNA-seq have deciphered TAM heterogeneity across various cancer types. Cheng et al. generated a single-cell atlas of tumor-infiltrating myeloid cells across different cancers, and revealed that each cancer type expressed different heterogeneous macrophage transcriptomic patterns [115]. Importantly, they further discovered the co-expression of M1 and M2 signatures in almost all cancer types, challenging the classical binary model of TAM and TAM polarization toward M2-like phenotypes [115]. Their findings imply the limitation of approaching TAMs via the classical binary M1/M2 concept as well as perceiving TAMs as a dominantly M2-like polarized population. Furthermore, Ma et al. also used SC RNA-seq to delineate seven distinct TAM subtypes across all cancer types [116]. These subtypes include interferon-primed, immune regulatory, inflammatory cytokine-enriched, lipid-associated, proangiogenic, resident-tissue-like, and proliferating TAMs. Consequently, the authors further proposed a new consensus TAM subset model based on this diversity, rather than the classical binary M1/M2 model.

Such co-expression of M1 and M2 features and the diversity of TAM subtypes was also asserted by Li et al. in CC [117]. Through SC RNA-seq, they investigated the immune subpopulations across different stages of CC progression, including normal cervix, premalignant lesion (high-grade squamous intraepithelial lesion, HSIL), primary cervical tumors, and metastatic lymph nodes. Intriguingly, they identified seven distinct macrophage clusters, expanding upon the simple binary classification. Using gene set variation analysis, they characterized cluster 1 (C1) and cluster 2 (C2) as macrophage groups with prominent immunomodulatory functions among the seven clusters. Notably, C1 comprised macrophages within tumor tissue exhibiting M2-like features such as a high immune escape score while C2 comprised M1-like macrophages with a high anti-inflammatory score. Furthermore, both M1 and M2 gene markers were upregulated in C1, suggesting the coexistence of M1 and M2 phenotypes within the TME. Other clusters also exhibited features closely associated with cancer progression: C6 displayed inflammation suppression near blood vessels, C5 regulated the lymph node microenvironment, and C8 exhibited highly regulated genes related to tumor cell proliferation, migration, and EMT [117]. Altogether, Li et al. emphasized the coexistence of M1 and M2 TAMs in the TME and highlighted the complexity of TAM subtypes beyond the traditional dichotomy concept. 

Similarly, Yue et al. also identified several subclusters of macrophages in their analysis and emphasized the necessity of using SC RNA-seq to explore the intricate cellular and functional heterogeneity of the TME in CC [118]. Moreover, Li et al. employed a multi-omics approach and identified novel TAM gene signatures that effectively distinguished subgroups of patients with CC based on prognosis and tumor stage, outperforming the traditional M1/M2 dichotomy and emphasizing the necessity for a more nuanced understanding of TAMs in CC via a multi-omics approach [119].

SP RNA-seq is another promising investigative tool that enables a comprehensive understanding of TAM dynamic interplay within the TME by incorporating spatial and location-wise knowledge [120,121,122]. Unlike SC RNA-seq, which necessitates the dissociation of cells from the original tissue, SP RNA-seq enables researchers to capture gene expression data directly from intact tissue sections, preserving the original physiological context [123,124]. By retaining spatial localization and distribution information, SP RNA-seq facilitates the consideration of tissue architecture and enables the digital profiling of cell identity and function. These characteristics of SP RNA-seq allow for the in-depth investigation of TAMs and their interactions with neighboring cell types such as tumor cells or T cells within the TME. 

A review by Wu et al. underscores the necessity of redefining the TAM subpopulation within the TME by integrating multi-omics approaches via techniques such as SC RNA-seq or SP RNA-seq [125]. They argue that varying prognosis outcomes associated with TAMs across different cancer types highlights the inter- and intra-heterogeneity of TAMs, influenced by distinct activation status and, importantly, different spatial localizations within the TME of each cancer type. They further emphasized that the specific spatial distribution of TAMs may exert consequential effects on tumor growth and prognosis, stressing the critical importance of integrating temporal and spatial information to elucidate the role of TAMs within the TME [125]. Grovel et al. employed SP RNA-seq methods in their study and emphasized the significance of considering the spatial information of immune cells within the TME in CC [126]. They highlighted the crucial role of accounting for the location of immune cells within the TME, rather than solely relying on their presence or abundance to enhance the interpretation of reciprocal immune cell interactions. However, despite the importance of TAM research using SP RNA-seq, few studies have adopted SP RNA-seq in CC research, which highlights the necessity of such an approach even more.

These findings regarding TAMs via SC RNA-seq and SP RNA-seq techniques challenge the classical dichotomy in understanding TAMs. Furthermore, this demonstrates the necessity of employing these new technologies to provide novel insights and possibly redefine our current understanding of TAMs in CC. Furthermore, integration results from SC RNA-seq and SP RNA-seq into TAM-targeted therapies would minimize off-target side effects, enhance the therapeutic efficacy, and further facilitate safer and better treatment responses in patients with CC, ultimately leading to better outcomes.

## 6. Conclusions

Despite their critical role and significance, the involvement of TAMs in CC has been less investigated compared with that in other gynecological cancers such as ovarian or endometrial cancer. However, TAMs are actively engaged in the TME of CC, contributing to almost every hallmark of cancer, including tumor cell proliferation, tumoral angiogenesis, invasion, or metastasis. With the development and advancement of research methodologies, recent research findings are focusing on a new understanding and conceptualization of TAMs beyond the traditional binary M1/M2 TAM classification. Therefore, this review emphasizes the necessity and importance of the active investigation of TAMs in CC with SC RNA-seq and SP RNA-seq to enable a more precise, in-depth, and holistic understanding of TAMs in CC that would facilitate the development of TAM-targeted and personalized immunotherapy approaches and ultimately improve treatment outcomes of patients with CC.
